# First Report of a Multidrug-Resistant *Mammaliicoccus sciuri* Strain Harboring *exhB* Isolated from an Alpaca with an Intracranial Abscess

**DOI:** 10.3390/vetsci13080836

**Published:** 2026-08-20

**Authors:** Wei Dong, Aobo Zhang, Ruiting Che, Nana Peng, Zhangquan Zhou, Junhao Liang, Meng Ge, Lei Yang

**Affiliations:** College of Veterinary Medicine, Hunan Agricultural University, Changsha 410128, China; dongwei@hunau.edu.cn (W.D.); 1191692902@stu.hunau.edu.cn (A.Z.); ruitingche@stu.hunau.edu.cn (R.C.); pengnana@stu.hunau.edu.cn (N.P.); 1441680391@stu.hunau.edu.cn (Z.Z.); 2231620928@stu.hunau.edu.cn (J.L.)

**Keywords:** alpaca, otitis media, intracranial abscess, *Mammaliicoccus sciuri*, *exhB*

## Abstract

Intracranial abscesses are extremely rare in alpacas, and previously reported cases have lacked systematic etiological characterization. The virulence characteristics of *Mammaliicoccus sciuri* (formerly *Staphylococcus sciuri*) in alpaca intracranial infections remain unclear. We investigated an alpaca with chronic otitis media and an intracranial abscess. Bacteria were recovered from intracranial purulent material and several other sites. A representative isolate was designated CS234. It was identified as *M. sciuri* by 16S rDNA sequencing and whole-genome sequencing (WGS). Average nucleotide identity (ANI) analysis showed 95.58% identity with *M. sciuri* NCTC12103. WGS identified *mecA*, *mecA1*, *fexA*, *optrA*, and *sal(A)*. The isolate showed phenotypic resistance to aminoglycosides, tetracyclines, and macrolides. PCR screening detected several virulence-associated gene targets, including *exhB* and tsst-1. To our knowledge, this is the first report of *exhB* in *M. sciuri*. In an intraperitoneal mouse challenge, the median lethal dose (LD_50_) was 4.4 × 10^7^ colony-forming units (CFU)/g body weight. Multiple-organ lesions were also observed. These findings indicate systemic pathogenic potential in mice. Recovery of *M. sciuri* from several affected sites indicates a strong etiological association with the suppurative disease in this alpaca. These findings expand current knowledge of *M. sciuri* infection and antimicrobial resistance in alpacas. They may also aid the diagnosis and treatment of similar cases.

## Simple Summary

Intracranial abscesses (collections of pus inside the skull) are extremely rare in alpacas, and little is known about the bacteria associated with these infections. In this study, we investigated an alpaca with an intracranial abscess and a chronic ear infection. Bacterial colonies with similar morphology were isolated from several sites, including the brain, ear, and blood. A representative isolate, designated CS234, was identified as *Mammaliicoccus sciuri*, a bacterium commonly found on the skin and mucosa of animals and humans. The isolate was resistant to several commonly used antibiotics and carried a virulence-associated gene that has not previously been reported in this species. In an intraperitoneal mouse challenge, the isolate caused lesions in several organs, including pulmonary congestion and splenic damage. These findings indicate systemic pathogenic potential in mice. The recovery of *M. sciuri* from several affected sites supports a strong etiological association with the suppurative disease in this alpaca. This study provides useful information for the diagnosis and antimicrobial treatment of similar infections in alpacas.

## 1. Introduction

Intracranial abscesses and other space-occupying intracranial lesions are rarely reported in alpacas (Vicugna pacos) [1,2,3]. They may be caused by bacteria, fungi, or parasites; however, bacterial infections are the primary cause of neurological disease in most cases [4]. In previous studies, bacterial pathogens associated with intracranial infections have included *Pseudomonas aeruginosa* [5], *Streptococcus* spp. [6], *Trueperella pyogenes* [7], *Listeria* spp. [8], *Fusobacterium necrophorum* [9], and *Staphylococcus aureus* [10]. Intracranial abscesses typically arise from direct extension of primary head infections (e.g., nasal passages, middle ear, or head wounds) or from hematogenous dissemination resulting from systemic infections [3,6,11]. Chronic suppurative otitis media can cause both extracranial and intracranial complications. In severe cases, it may lead to intracranial abscess and death, especially when cholesteatoma is present [12]. The long, narrow, and sigmoidal external ear canal may predispose alpacas to otitis media [13]. In alpacas, extension of otitis media into adjacent intracranial structures has been proposed as a possible route of infection, but the causative pathogen was not identified in the previously reported case of an otitis-associated intracranial abscess.

*Mammaliicoccus sciuri* is a coagulase-negative member of the family Staphylococcaceae and a common commensal of the skin and mucosa of various animals and humans [14,15,16,17,18]. The *M. sciuri* group is considered an important reservoir of antimicrobial resistance determinants, including *mecA*. This gene can be carried by *M. sciuri* and may be transferred among staphylococci through staphylococcal cassette chromosome mec (SCCmec) elements [19,20]. *M. sciuri* can also harbor various virulence-associated genes homologous to those found in other staphylococci, some of which may have been acquired through horizontal gene transfer [21]. Given that *M. sciuri* occurs in both animal and human hosts, the potential horizontal transfer of such virulence-associated genes among bacteria at the livestock–human interface warrants attention. Among these, exhB is of particular interest because it encodes exfoliative toxin B in Staphylococcus hyicus [22]. However, it remains unclear whether exhB in *M. sciuri* is associated with mobile genetic elements or has the potential for horizontal transfer among host-associated bacterial populations. In recent years, clinical infections associated with *M. sciuri* have increasingly been reported in pigs, horses, gorillas, and humans [23,24,25,26]. In contrast, information on this bacterium in alpacas remains limited. In a passive surveillance study of 99 alpacas, methicillin-resistant coagulase-negative staphylococci and mammaliicocci were isolated from 10.1% of nasal swabs, and *M. sciuri* was recovered from 7.1% (7/99) of the animals [27]. These findings indicate that *M. sciuri* can occur in alpaca populations, but its pathogenic characteristics and antimicrobial resistance profile in alpaca intracranial infections remain unclear.

In March 2023, a captive alpaca in a wildlife park in Hunan Province, China, developed chronic left-sided otitis media accompanied by progressive neurological signs (head tilt, torticollis, drowsiness, and paralysis). Due to the poor prognosis, the animal was humanely euthanized and submitted for necropsy. We hypothesized that a bacterial agent was associated with the chronic otitis media and intracranial abscess in this alpaca. We also hypothesized that the recovered isolate might harbor antimicrobial resistance determinants and virulence-associated determinants and show systemic pathogenic potential in mice. To test these hypotheses, we performed bacterial isolation and identification, 16S rDNA sequencing, whole-genome sequencing, antimicrobial susceptibility testing, polymerase chain reaction (PCR) screening for antimicrobial resistance and virulence-associated genes, and an intraperitoneal mouse challenge.

## 2. Materials and Methods

### 2.1. Necropsy and Sample Collection

A complete necropsy was performed on the alpaca, and samples of ear canal exudate, peripheral blood, brain parenchyma, left ear lymph nodes, and intracranial purulent material were aseptically collected for bacteriological examination.

### 2.2. Experimental Animals

Female ICR mice (6 weeks old; body weight, 20 ± 2 g) were obtained from Hunan SJA Laboratory Animal Co., Ltd. (Changsha, China). Mice were housed under standard specific-pathogen-free conditions with ad libitum access to food and water and were acclimatized before experiments.

### 2.3. Isolation and Culture of Bacteria

Peripheral blood, ear canal exudate, intracranial purulent material, brain tissue, and left ear lymph nodes were aseptically collected. These samples were inoculated onto fresh blood agar plates and incubated at 37 °C for 18 h. Single colonies were selected for Gram staining and purified in Luria–Bertani (LB) broth.

### 2.4. 16S rDNA Sequencing, Whole-Genome Sequencing, and Bioinformatic Analysis

Universal primers 16S rDNA-27F (5′-AGAGTTTGATCCTGGCTCAG-3′) and 16S rDNA-1492R (5′-GGCTACCTTGTTACGACTT-3′) were synthesized by Beijing Tsingke Biotechnology Co., Ltd. (Beijing, China). Each 50 μL PCR mixture contained 1 μL of forward primer (10 μmol/L), 1 μL of reverse primer (10 μmol/L), 25 μL of 2× KeyPo Master Mix (Dye Plus; Vazyme Biotech Co., Ltd., Nanjing, China), 2 μL of DNA template (50 ng/μL), and nuclease-free water to a final volume of 50 μL. The amplification conditions were as follows: 98 °C for 1 min; 35 cycles of 98 °C for 10 s, 56 °C for 5 s, and 72 °C for 10 s; followed by 72 °C for 10 min. PCR products (8 μL) were analyzed by 1% agarose gel electrophoresis, and positive amplicons were sequenced by Beijing Tsingke Biotechnology Co., Ltd.

Based on the 16S rDNA sequencing result, the isolate was subjected to whole-genome sequencing (WGS). Genomic DNA extraction, library preparation, and sequencing were performed by Beijing Tsingke Biotechnology Co., Ltd. (Beijing, China). Sequencing was conducted using the Oxford Nanopore MinION platform (Oxford Nanopore Technologies plc, Oxford, UK). The filtered reads were assembled using Wtdbg2 v2.5. The assembly was polished with Racon v1.5.0 and circularized using Circlator v1.5.5. This workflow was selected for the assembly and refinement of the Oxford Nanopore long-read sequencing data.

Genome completeness was evaluated using CheckM v1.1.3 and BUSCO v5.8.2. Protein-coding genes were predicted using Prodigal v2.6.3. tRNA and rRNA genes were predicted using tRNAscan-SE v2.0.9 and Infernal v1.1.3, respectively. Genome annotation was also performed using the National Center for Biotechnology Information (NCBI) Prokaryotic Genome Annotation Pipeline (PGAP v6.11). Species-level identification was assessed using FastANI v1.34 against the genome of *M. sciuri* NCTC12103.

Antimicrobial resistance determinants were screened using the Comprehensive Antibiotic Resistance Database/Resistance Gene Identifier (CARD/RGI v6.0.8) and ResFinder 4.7.2. Virulence-associated loci were screened using VFanalyzer. Because a Mammaliicoccus-specific reference dataset was unavailable in VFanalyzer, the Staphylococcus framework was used for exploratory analysis. Known plasmid replicons were screened using PlasmidFinder 2.1, and SCCmec-associated elements were analyzed using SCCmecFinder 1.2. Genomic islands and prophage regions were predicted using IslandPath-DIMOB v0.2 and PhiSpy v2.3, respectively.

The 16S rDNA sequence of CS234 was aligned with reference sequences of *M. sciuri* and related staphylococcal species retrieved from GenBank using MEGA11. A phylogenetic tree was constructed using the neighbor-joining method based on the Kimura two-parameter model, and branch support was evaluated using 1000 bootstrap replicates.

### 2.5. Biochemical Tests

The trace biochemical reaction tube (Hangzhou Microbial Reagent Co., Ltd., Hangzhou, China) was selected based on Bergey’s Manual of Systematic Bacteriology, 2nd edition, Volume 3: The Firmicutes (2009) [28]. The isolate was seeded into the LB liquid culture medium at 37 °C for 12 h. The culture medium was then seeded into the selected trace biochemical identification tubes at 37 °C for 48 h, followed by observation and analysis of experimental results.

### 2.6. Antimicrobial Susceptibility Testing and Antimicrobial Resistance Gene Detection

The antimicrobial susceptibility of the isolate was tested by the disk diffusion method on Mueller-Hinton agar using commercial antimicrobial disks (Hangzhou Microbial Reagent Co., Ltd., Hangzhou, China). Disk diffusion was selected for broad initial phenotypic screening across multiple antimicrobial classes. Plates were incubated at 37 °C for 18 h. This incubation time was within the 16–20 h range specified by the Clinical and Laboratory Standards Institute (CLSI) criteria used in this study. Inhibition zone diameters were then recorded. Results were interpreted using CLSI M100, 33rd edition (2023). Because specific breakpoints for *M. sciuri* were unavailable, the criteria for Staphylococcus spp. were used as surrogate criteria when applicable. For antimicrobial agents without applicable criteria, only the inhibition zone diameters were reported. No S/I/R category was assigned to these agents. Minimum inhibitory concentration (MIC) testing was not performed.

For the detection of resistance genes, bacterial DNA was extracted using the boiling method. Subsequently, PCR amplification of specific resistance genes was performed using primers listed in Table 1 [29]. The PCR mixture and cycling conditions were the same as those described for 16S rDNA amplification in Section 2.4, except that the annealing temperature was adjusted for each primer pair as listed in Table 1. PCR products were analyzed by 1% agarose gel electrophoresis as described in Section 2.4. All primers were synthesized by Beijing Tsingke Biotechnology Co., Ltd.

### 2.7. PCR Screening of Virulence-Associated Genes

Primer sequences and annealing temperatures are listed in Table 2 [30]. Template DNA was extracted using the boiling method. PCR was used to screen for virulence-associated gene targets, including coagulase (*coa*), α-hemolysin (*hla*), β-hemolysin (*hlb*), δ-hemolysin (*hld*), γ-hemolysin (*hlg*), fibrinogen-binding protein (*fib*), fibronectin-binding proteins (*fnbA* and *fnbB*), clumping factor A (*clfA*), accessory gene regulator (*agr*), capsular polysaccharide type 5 (*cap5*), toxic shock syndrome toxin-1 (*tsst-1*), exfoliative toxins (*exhA*, *exhB*, and *exhC*), and Panton–Valentine leukocidin (pvl) in the isolated strain. The PCR mixture and cycling conditions were the same as those described for 16S rDNA amplification in Section 2.4, except that the annealing temperature was adjusted for each primer pair as listed in Table 2. PCR products were analyzed by 1% agarose gel electrophoresis as described in Section 2.4. The *exhB* PCR product was further examined by bidirectional Sanger sequencing. All primers were synthesized by Beijing Tsingke Biotechnology Co., Ltd.

### 2.8. Intraperitoneal Mouse Challenge

The purified isolate was cultured in LB broth at 37 °C for 12 h, and the bacterial concentration was determined by plate counting. Bacterial cells were then harvested, washed, and resuspended in sterile saline to prepare inocula at the required concentrations. Sterile saline was used as the vehicle control. The intraperitoneal route was used to estimate the median lethal dose (LD_50_) and assess systemic pathogenicity. This route provided a controlled systemic challenge. It was not intended to reproduce the natural spread of infection from the middle ear to the cranial cavity. It was also not used to assess neurovirulence or brain abscess formation.

Pilot experiment: Because no previous LD_50_ data were available for this isolate, a broad dose range was used for initial range finding. Forty-eight ICR mice were randomly assigned to eight groups (*n* = 6/group). Seven groups received 0.2 mL of bacterial suspension by intraperitoneal injection at 1.00 × 10^7^, 2.00 × 10^7^, 4.00 × 10^7^, 6.00 × 10^7^, 8.00 × 10^7^, 1.00 × 10^8^, or 1.20 × 10^8^ CFU/g body weight. One group received sterile saline. Dn was defined as the highest dose that caused no mortality during the observation period. Dm was defined as the lowest dose that caused 100% mortality. These values were used to determine the dose range for the formal LD_50_ experiment.

Formal LD_50_ experiment: Sixty ICR mice were randomly assigned to six groups (*n* = 10/group) using a random number method and challenged intraperitoneally with graded doses based on Dn and Dm. The six challenge doses were spaced geometrically between Dn and Dm. The dose ratio was calculated as r = (Dm/Dn)^1/(k−1)^, where k was the number of dose groups. Mice were monitored at least twice daily for 7 days. Humane endpoints included severe lethargy, inability to access food or water, hypothermia, and signs of unrelieved pain. Mice that met any one of these criteria were euthanized immediately as described in Section 2.9. Necropsy was performed under aseptic conditions; selected tissues were collected for bacteriological re-isolation (Section 2.3) and histopathology. Tissue samples were fixed in 10% paraformaldehyde and embedded in paraffin. Sections were stained with hematoxylin and eosin (H&E) and examined under a light microscope for pathological changes. Histopathological evaluation was performed in a non-blinded manner. Mortality data were analyzed using probit regression in IBM SPSS Statistics 27.0 (IBM Corp., Armonk, NY, USA). The relationship between mortality probability (p) and dose (D) was expressed as: Φ^−1^(p) = α + β log_10_(D), where Φ^−1^ is the inverse standard normal cumulative distribution function, and α and β are the fitted intercept and slope, respectively. At p = 0.5, the LD_50_ was calculated as: LD_50_ = 10^−α/β^.

The 95% fiducial confidence limits of the LD_50_ were obtained from the SPSS Probit procedure. For clarity and comparability with previous reports, challenge doses and LD_50_ values are presented primarily as CFU/mouse. Equivalent CFU/g body weight values were calculated using a standard mouse body weight of 20 g.

### 2.9. Ethics Statement

All procedures involving the alpaca and mice were reviewed and approved by the Ethics and Use of Laboratory Animals Committee of Hunan Agricultural University (approval no. 2024132; approved on 3 January 2024). Animal procedures were conducted in accordance with the national “Regulations on the Management of Experimental Animals” and the Experimental Animal Care and Use Guidelines of the Ministry of Science and Technology of China (MOST-2011-02). The study was reported in accordance with the ARRIVE 2.0 recommendations.

The alpaca was humanely euthanized after unsuccessful clinical treatment and because of the poor prognosis. Euthanasia was performed by intravenous administration of a lethal dose of pentobarbital sodium (approximately 100 mg/kg body weight) in accordance with institutional veterinary procedures.

Mice were monitored at least twice daily. Clinical condition was evaluated using four criteria: activity, ability to access food and water, body temperature, and signs of pain or distress. Each criterion was graded from 0 to 3. A score of 0 indicated normal condition. A score of 1 indicated a mild abnormality. A score of 2 indicated a moderate abnormality and required increased monitoring. A score of 3 indicated a severe abnormality and was considered a humane endpoint. A score of 3 in any single category resulted in immediate euthanasia.

Humane endpoint criteria included severe lethargy or markedly reduced responsiveness, inability to access food or water, severe hypothermia, and persistent signs of unrelieved pain. Mice that reached any of these endpoints were euthanized immediately by intraperitoneal administration of pentobarbital sodium at 150 mg/kg body weight. Cervical dislocation was then performed to confirm death.

## 3. Results

### 3.1. Bacterial Isolation and Identification

**Figure 1:**
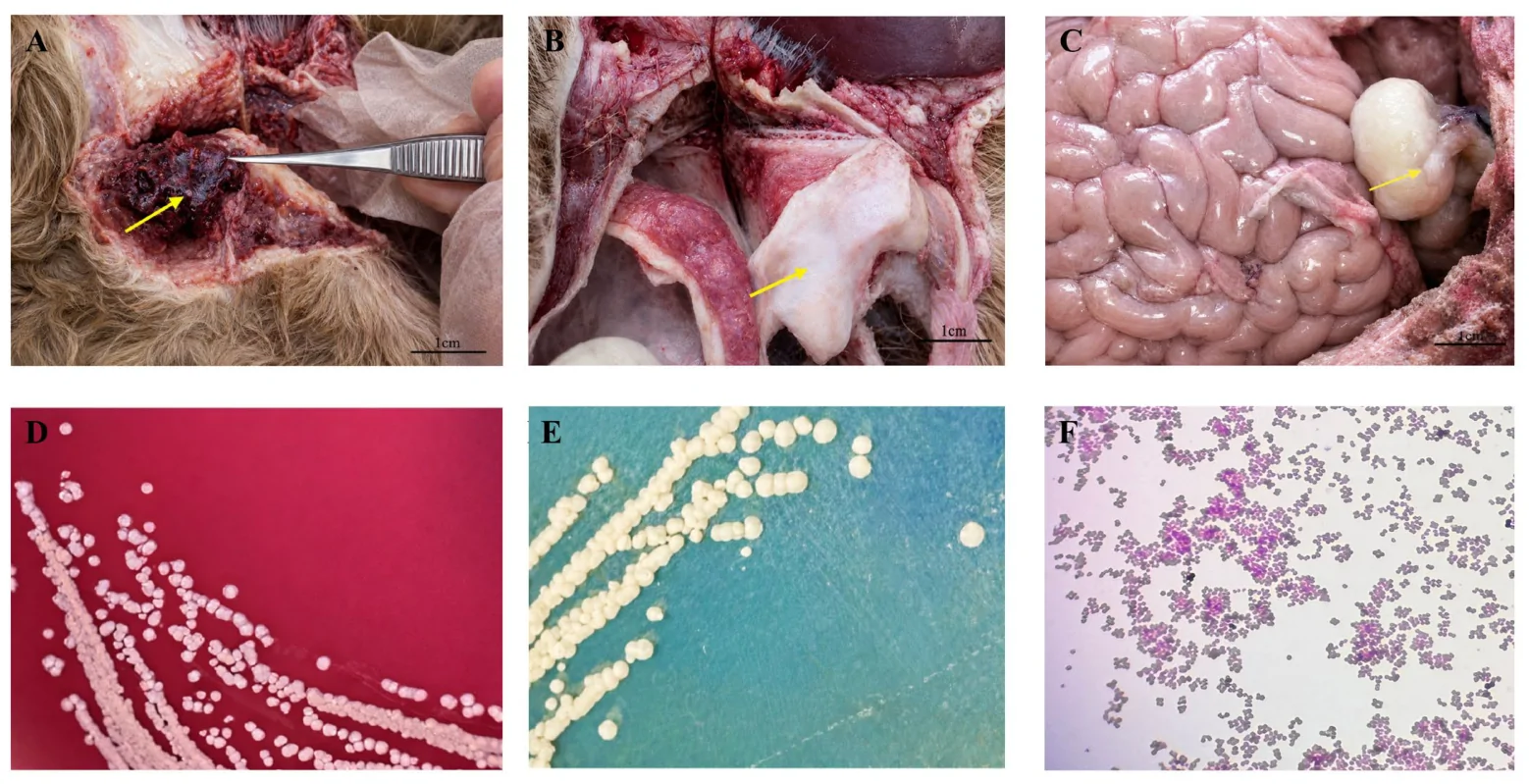
Pathological findings and bacterial morphology in the alpaca. (**A**–**C**) Gross pathological findings. (**A**) Enlarged and hemorrhagic left periauricular lymph node with fibrin on the surface (yellow arrow). (**B**) Purulent exudate in the tympanic cavity (yellow arrow). (**C**) Encapsulated purulent mass in the subarachnoid space ventral to the cerebellum (yellow arrow). The mass measured approximately 3 cm × 2 cm × 2 cm and compressed the midbrain and cerebellum. Scale bars in (**A**–**C**) = 1 cm. (**D**–**F**) Morphology of the bacterial isolate. (**D**) Non-hemolytic colonies on blood agar. (E) Yellow colonies on LB agar. (**F**) Gram-positive cocci in grape-like clusters.

Necropsy was performed to characterize the major lesions in the alpaca. Gross findings revealed that the left periauricular lymph node was enlarged and hemorrhagic, with fibrin on the surface of the lymph node (Figure 1A). Abundant white purulent exudate was observed in the tympanic cavity (Figure 1B). In addition, an encapsulated purulent mass was present in the subarachnoid space ventral to the cerebellum, measuring approximately 3 cm × 2 cm × 2 cm (Figure 1C).

Bacterial colonies with identical morphological characteristics were isolated from peripheral blood, intracranial purulent material, ear canal exudate, brain tissue, and the left periauricular lymph node. On blood agar, the colonies were creamy-white, circular, and non-hemolytic (Figure 1D). On LB agar, they appeared smooth, yellow, circular, and had entire margins (Figure 1E). Gram staining revealed purple, grape-like clusters of cocci, indicating that the isolate was a Gram-positive bacterium (Figure 1F).

### 3.2. 16S rDNA and Genome-Based Species Identification

**Figure 2:**
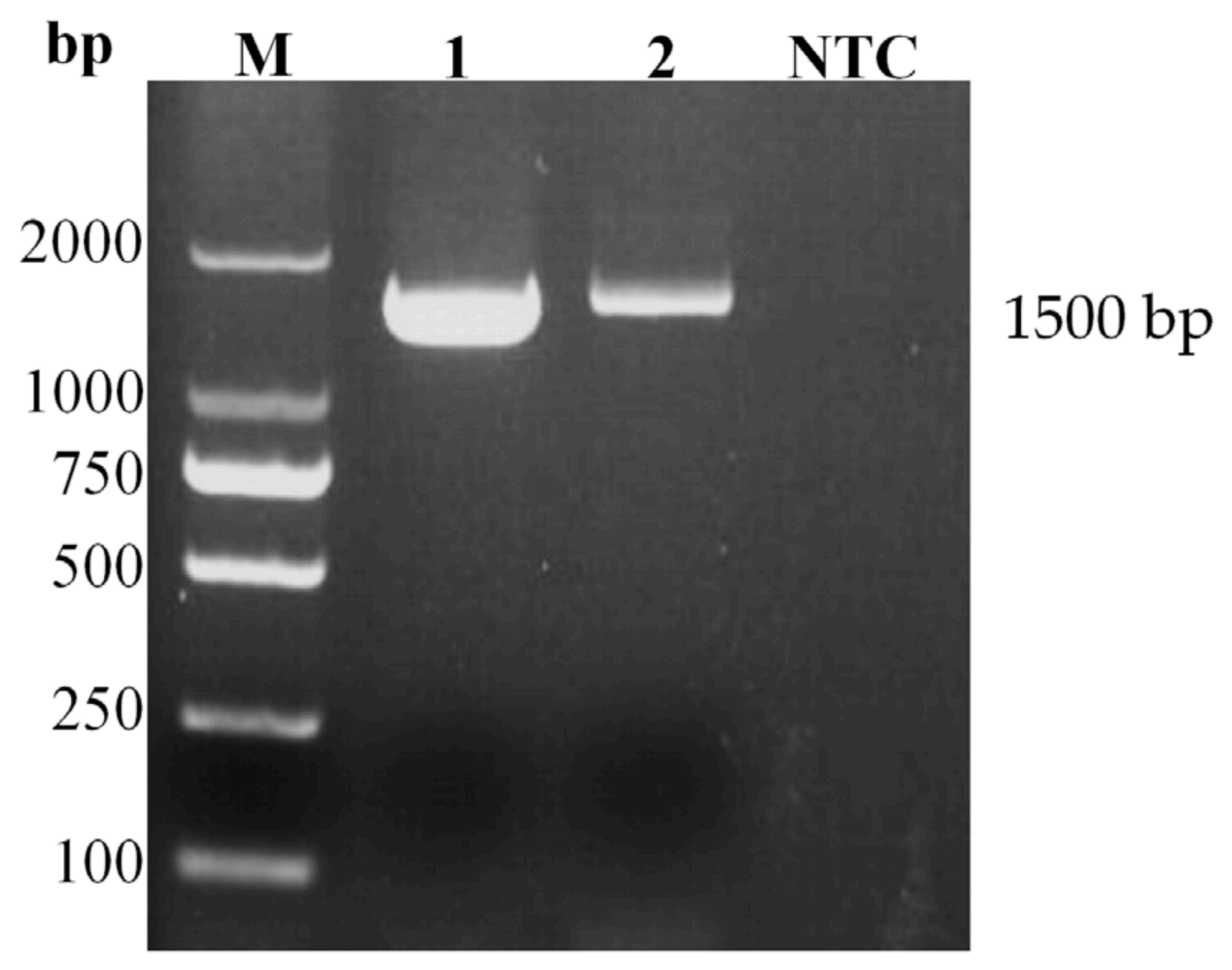
16S rDNA amplification product electrophoresis. M: DL5000 DNA marker; 1: 16S rDNA amplification product of the isolate from intracranial purulent material; 2: 16S rDNA amplification product of the isolate from the left periauricular lymph node; NTC: non-template control (no DNA added). No band of the expected target size was observed in the NTC lane.

**Figure 3:**
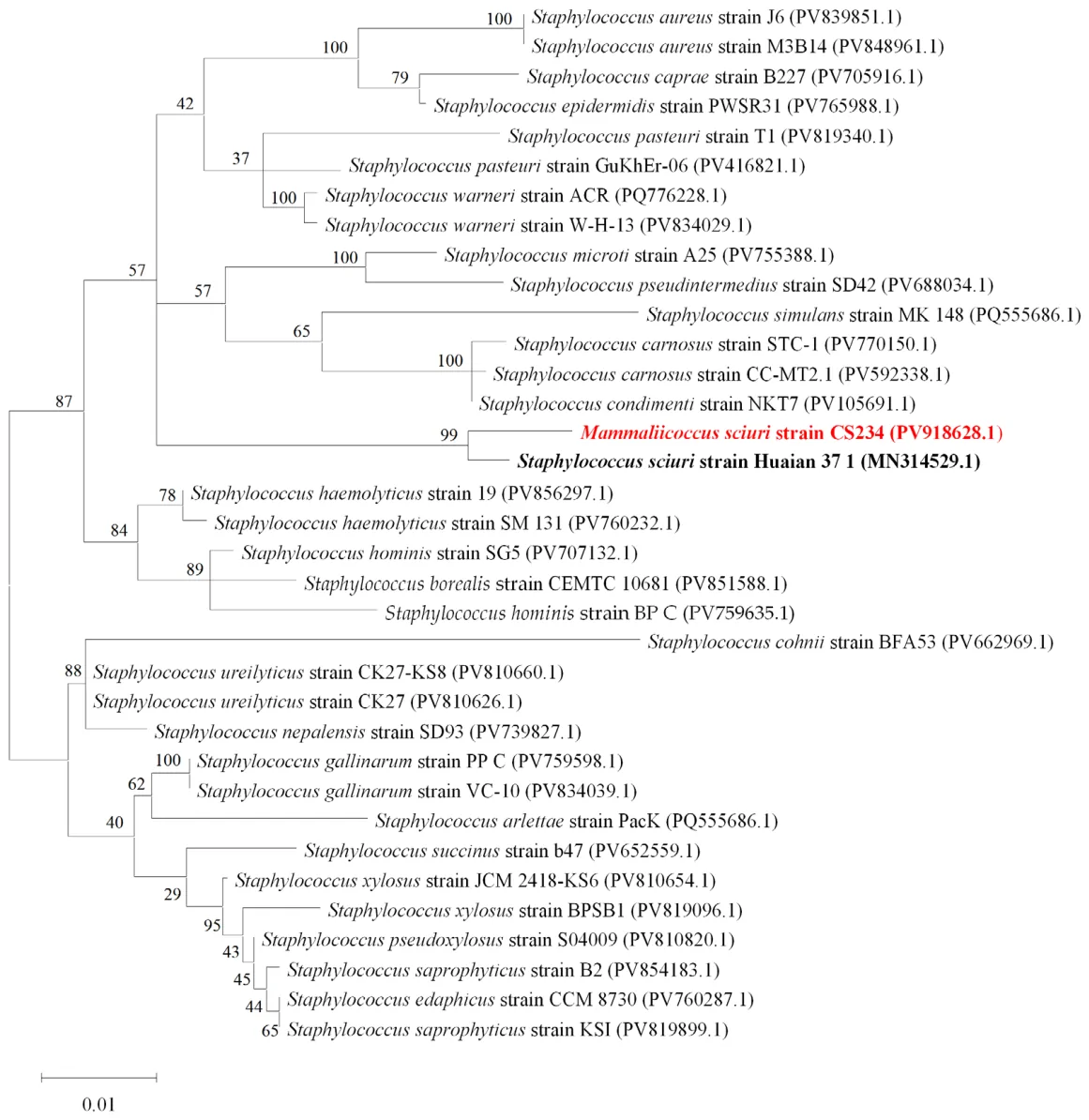
Phylogenetic analysis based on 16S rDNA sequences of the isolate and related staphylococcal species. The sequence of M. sciuri strain CS234 (PV918628.1) obtained in this study is highlighted in red and bold.

Using genomic DNA from the isolate as the template, amplification with universal 16S rDNA primers yielded a product of approximately 1500 bp (Figure 2). BLAST v2.17.0 analysis in NCBI showed 98.60% sequence identity with *Mammaliicoccus sciuri* (GenBank accession no. MN314529.1). Phylogenetic analysis showed that the isolate sequence (PV918628.1), deposited as *M. sciuri* strain CS234, clustered with *M. sciuri* (MN314529.1) (Figure 3). Because 16S rDNA sequence identity alone was insufficient for species-level confirmation, whole-genome sequencing was subsequently performed. The whole-genome sequence is available in GenBank under accession number CM152820.1. The GenBank record retains the original submission alias Sp-0816, which refers to the same isolate as CS234. FastANI analysis showed that strain CS234 shared 95.58% average nucleotide identity with *M. sciuri* NCTC12103. The isolate is referred to as CS234 throughout this manuscript.

### 3.3. Whole-Genome Characteristics

Whole-genome sequencing generated a 2.87-Mb genome with a GC content of 32.82%. The assembly consisted of two contigs and showed high completeness. Detailed assembly, coverage, and annotation statistics are summarized in Table 3.

### 3.4. Biochemical Characteristics

The isolate showed positive fermentation reactions for glucose, sucrose, mannitol, and maltose, but was negative for lactose. The citrate test was positive, whereas the Voges-Proskauer (V-P), indole, Methyl Red (MR), and hydrogen sulfide production tests were negative. Compared with the typical biochemical profile of *Mammaliicoccus sciuri*, CS234 showed consistent reactions for glucose, mannitol, sucrose, and the V-P test. Maltose and lactose reactions were within the reported variable characteristics of *M. sciuri* (Table 4). Overall, the biochemical characteristics of CS234 were consistent with the typical profile of *M. sciuri*.

### 3.5. Antimicrobial Susceptibility and Antimicrobial Resistance Determinants

**Figure 4:**
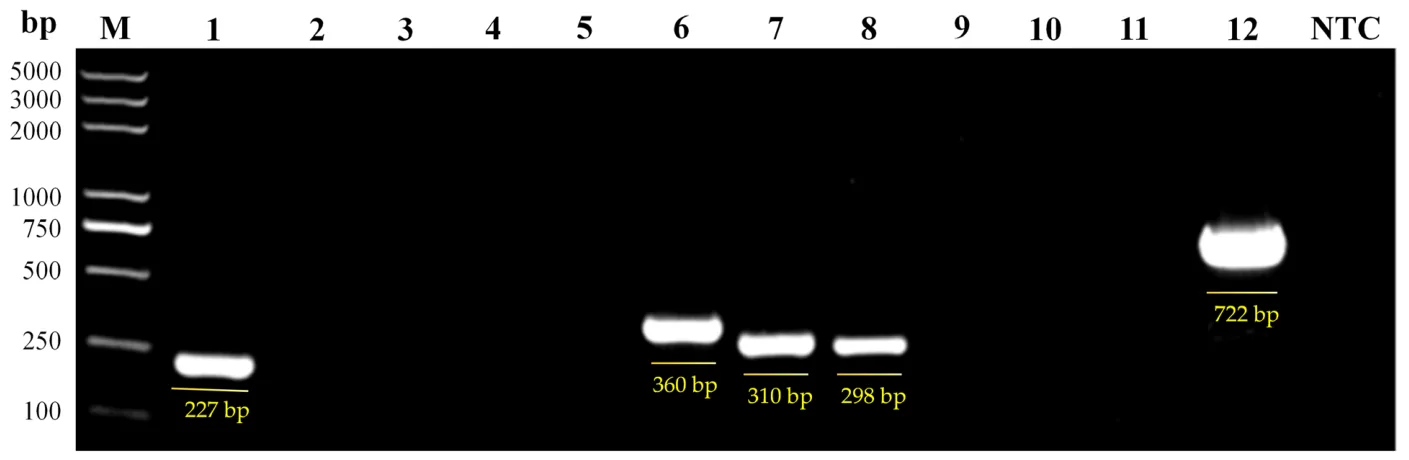
Electrophoresis of drug resistance gene amplification products of isolated strain. M, DL5000 DNA marker; lane 1, *aacA-aphD*; lane 2, *aphA3*; lane 3, *aadD*; lane 4, *ermA*; lane 5, *ermC*; lane 6, *tetK*; lane 7, *mecA*; lane 8, *mecA1*; lane 9, *mecC*; lane 10, *blaZ*; lane 11, *msr*; lane 12, *mphC*; NTC, non-template control (no DNA added). No band of the expected target size was observed in the NTC lane.

The isolate was resistant to penicillin G, gentamicin, tetracycline, doxycycline, and erythromycin. These agents represented several antimicrobial classes. For other agents without applicable surrogate disk diffusion criteria, only the inhibition zone diameters were reported (Table 5). The oxacillin inhibition zone was 17 mm. No categorical interpretation was assigned because an applicable *M. sciuri*-specific disk diffusion breakpoint was unavailable. PCR screening yielded target-sized amplicons for *aacA-aphD*, *tetK*, *mecA*, *mecA1*, and *mphC* (Figure 4).

ResFinder identified five acquired antimicrobial resistance determinants on Contig00001: *mecA*, *mecA1*, fexA, optrA, and sal(A). Their nucleotide identities were 100%, 99.90%, 99.65%, 100%, and 94.16%, respectively. All five hits showed 100% sequence coverage. CARD independently supported *mecA* and fexA and also identified the adjacent mecR1 and mecI regulatory genes.

SCCmecFinder identified a class A mec gene complex containing *mecA*, *mecR1*, and *mecI*. However, no complete recognized SCCmec type could be assigned. The mec gene complex was flanked by IS431mec-associated transposase genes.

In contrast, *aacA-aphD*, *tetK*, and *mphC* yielded amplicons of the expected sizes in the PCR screening, but these resistance determinants were not confirmed by CARD or ResFinder. In addition, minocycline showed an intermediate phenotype in the disk diffusion test. However, genome analysis did not identify any known resistance determinant that could clearly explain this phenotype.

### 3.6. Virulence-Associated Gene Detection and Genome-Based Analysis

**Figure 5:**
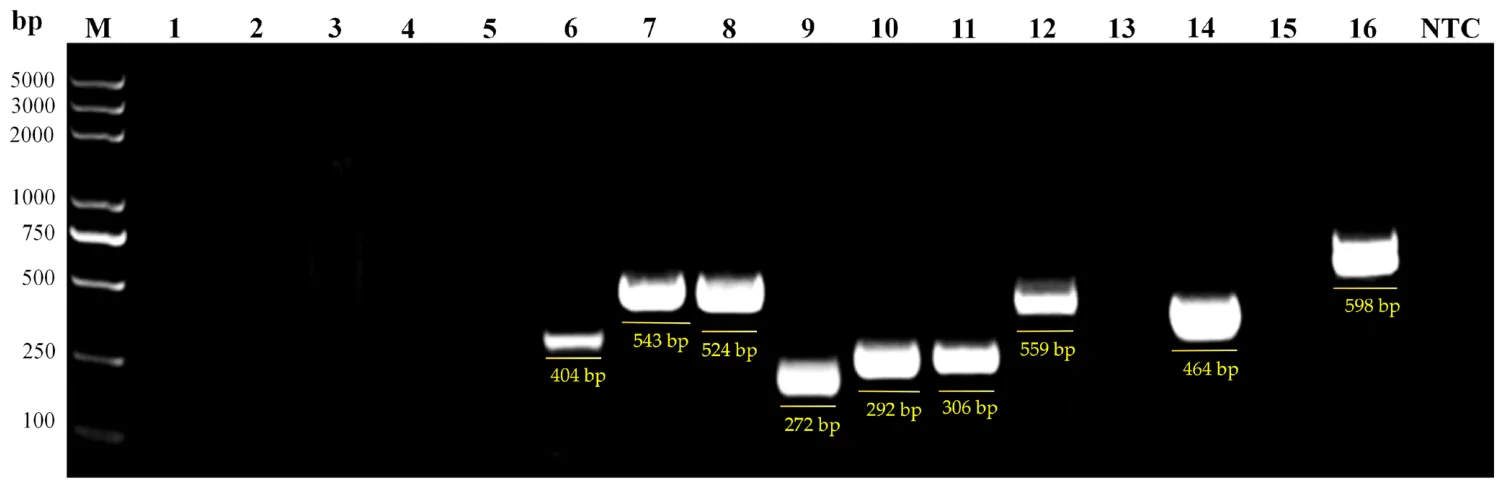
PCR amplification of virulence-associated genes in the *Mammaliicoccus sciuri* isolate. M, DL5000 DNA marker; lane 1, *coa*; lane 2, *hla*; lane 3, *hlb*; lane 4, *hld*; lane 5, *hlg*; lane 6, *fib*; lane 7, *fnbA*; lane 8, *fnbB*; lane 9, *clfA*; lane 10, *agr*; lane 11, *cap5*; lane 12, *tsst-1*; lane 13, *exhA*; lane 14, *exhB*; lane 15, *exhC*; lane 16, *pvl*; NTC, non-template control (no DNA added). No band of the expected target size was observed in the NTC lane.

PCR screening yielded target-sized amplicons for *fib*, *fnbA*, *fnbB*, *clfA*, *agr*, *exhB*, *pvl*, *tsst-1*, and *cap5*, whereas *coa*, *hla*, *hlb*, *hld*, *hlg*, *exhA*, and *exhC* were not detected (Figure 5). Bidirectional Sanger sequencing verified the sequence identity of the *exhB* amplicon.

VFanalyzer identified several putative virulence-associated loci in the CS234 genome. These included an ica-associated biofilm locus containing icaA, icaB, and icaC and an sspA-associated glutamyl endopeptidase. Putative homologs related to staphylococcal surface adhesion proteins were also detected. Notably, not all PCR-detected virulence-associated targets were independently supported by VFanalyzer. Because a Mammaliicoccus-specific reference dataset was unavailable, these in silico predictions were interpreted cautiously.

IslandPath-DIMOB predicted four genomic islands on Contig00001, and PhiSpy identified one prophage region. The 27,242-bp secondary contig was provisionally classified as plasmid-associated by the sequencing pipeline. PlasmidFinder did not identify any known plasmid replicons. The current analysis did not establish a genomic association between *exhB* and these predicted mobile genetic elements.

### 3.7. Results of the Intraperitoneal Mouse Challenge

Based on the Dm and Dn values, the bacterial group spacing ratio (r) was determined to be 1.585. The distribution of challenge doses and corresponding mortality data is presented in Table 6. No abnormalities were observed in the control group. In the infected groups, dead mice showed congestion, hemorrhage, and swelling in the lungs, spleen, and kidneys. No gross lesions were observed in the brain during necropsy. Colonies with morphology consistent with the inoculated strain were recovered from selected tissues of moribund or dead mice. No bacterial growth was observed in the control group.

**Figure 6:**
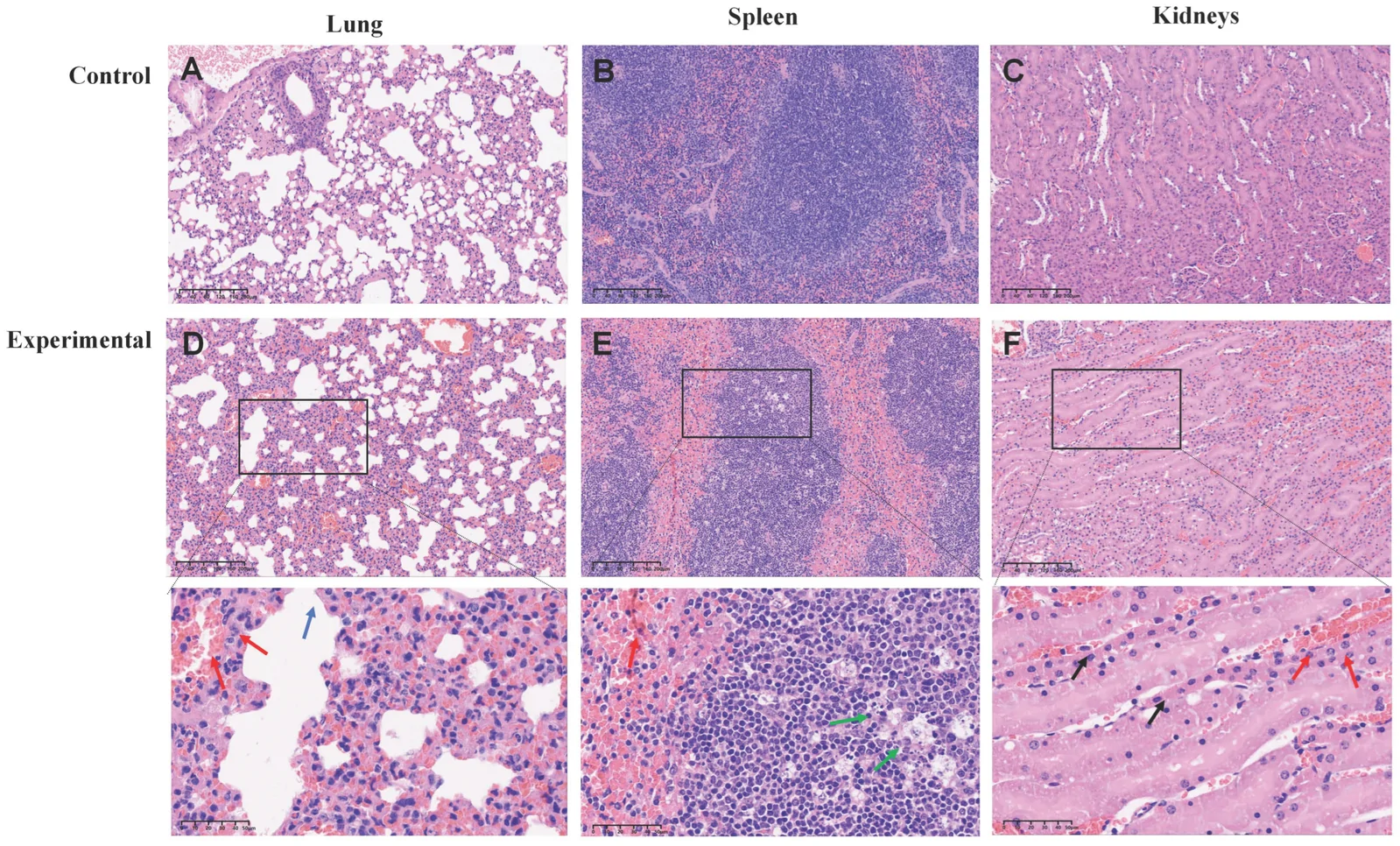
H&E-stained sections of the lungs, spleen, and kidneys from ICR mice. (**A**–**C**) Control group. No significant histopathological lesions were observed in the lung (**A**), spleen (**B**), or kidney (**C**). (**D**–**F**) Infected group. Boxed areas are shown at higher magnification below the corresponding panels. (**D**) Lung: thickening of the alveolar septa (blue arrows) and pulmonary congestion (red arrows). (**E**) Spleen: the white pulp exhibited reduced volume. Lymphocytes with apoptotic morphology showed cellular shrinkage and separation from surrounding cells (green arrows). Marked hemorrhage was also observed in the red pulp sinusoids (red arrows). (**F**) Kidney: renal tubular epithelial cells showed marked necrosis, including pyknosis, karyorrhexis, and karyolysis (black arrows). Diffuse hemorrhage was observed in the renal interstitium (red arrows).

The LD_50_ was 8.8 × 10^8^ CFU per mouse. This was equivalent to 4.40 × 10^7^ CFU/g body weight. The 95% confidence limits were 6.68 × 10^8^ to 1.23 × 10^9^ CFU per mouse. H&E staining showed lesions in the lungs, spleen, and kidneys of infected mice (Figure 6).

## 4. Discussion

Previous studies have identified several bacterial pathogens associated with otitis media, including *Pseudomonas aeruginosa*, *Staphylococcus aureus*, *Trueperella pyogenes*, and *Klebsiella* spp. [31]. Some of these bacteria can spread from the middle ear to adjacent structures and cause neurological disease. Câmara et al. reported a case of suppurative meningoencephalitis in a dairy cow associated with extension of P. aeruginosa otitis media toward the cranial cavity [5]. Riker et al. reported chronic otitis media in a kangaroo with an intracranial inflammatory polyp caused by *T. pyogenes* [7]. In alpacas, Franz et al. described an intracranial abscess secondary to otitis media, but the associated bacterial pathogen was not identified [3]. In the present case, severe suppurative lesions were observed in the left ear and cranial cavity. Their anatomical distribution and gross pathological features support the possibility of local extension from the middle ear into the cranial cavity. However, bacteria with the same colony morphology were also isolated from peripheral blood, so hematogenous dissemination cannot be excluded. The available evidence is insufficient to distinguish between these two routes. Bacteria with consistent colony morphology were recovered from several anatomically distinct affected sites, including intracranial purulent material, brain tissue, ear canal exudate, peripheral blood, and the left periauricular lymph node. This reduces the likelihood of incidental contamination from a single sample. A representative isolate was identified as *Mammaliicoccus sciuri* by 16S rDNA sequencing and whole-genome ANI analysis. These findings support a strong etiological association between *M. sciuri* and the suppurative disease in this alpaca. To our knowledge, this is the first identification of *M. sciuri* in an alpaca with otitis media complicated by an intracranial abscess.

*Mammaliicoccus sciuri* has long been regarded as a commensal organism of the skin and ear canal mucosa of animals and as a coagulase-negative opportunistic pathogen. Previous studies have shown that *M. sciuri* strains may carry various virulence-associated factors and can be isolated from different types of clinical infections. For example, *eno* and *agr* have been detected in isolates associated with bovine mastitis [32], *hla* and *hlg* in isolates from bacterial meningitis in ducks [33], and *exhC* in isolates associated with exudative epidermitis in piglets [34]. In the present study, PCR screening produced amplicons of the expected sizes for several virulence-associated gene targets. These included fibrinogen-binding protein (*fib*), fibronectin-binding proteins (*fnbA* and *fnbB*), clumping factor A (*clfA*), accessory gene regulator (*agr*), exfoliative toxin B (*exhB*), Panton–Valentine leukocidin (*pvl*), toxic shock syndrome toxin 1 (*tsst-1*), and capsular polysaccharide type 5 (*cap5*). These targets are associated with adhesion, regulation, toxin-related functions, and capsule formation [35], suggesting that CS234 has diverse potential infection-related genetic features. However, gene expression, protein production, and biological activity were not examined in this study. Their functional significance therefore requires further validation.

Notably, this study reports *exhB* in *M. sciuri* for the first time. In *Staphylococcus hyicus*, *exhB* encodes exfoliative toxin B, a known virulence factor associated with exudative epidermitis. ExhB is a serine protease that can cleave desmoglein-1 (Dsg1) in susceptible hosts and disrupt epithelial cell adhesion [36,37]. However, the functional significance of exhB in this strain remains unclear. Based on the known activity of ExhB in *S. hyicus*, we propose a testable hypothesis. If ExhB is expressed and biologically active in alpaca tissues, it may disrupt epithelial cell adhesion and impair the local epithelial barrier in the infected ear. This could facilitate local bacterial invasion and spread into deeper tissues. However, the current evidence does not demonstrate that ExhB contributed to intracranial abscess formation in this case. Further studies could assess exhB expression and ExhB protein activity and compare CS234 with an exhB-deficient mutant and a complemented strain in epithelial barrier assays or infection models that better reproduce otitis-associated spread.

In addition to its functional significance, the genetic context and potential mobility of exhB in CS234 remain unclear. To further assess the possibility of horizontal transfer, we analyzed putative mobile genetic elements in the CS234 genome. Several genomic islands and one prophage region were predicted. A secondary contig was provisionally classified as potentially plasmid-associated, but no known plasmid replicon was detected. The current analysis did not establish an association between *exhB* and these predicted mobile genetic elements. Therefore, the mobility of *exhB* in CS234 remains uncertain. Comparative genomic analyses of isolates from different host sources will be needed to determine whether *exhB* is associated with transferable genetic elements.

The intraperitoneal mouse challenge was used to evaluate the systemic pathogenic potential of CS234. Infection resulted in mortality and multiple-organ lesions, but no gross brain lesions were observed. These findings indicate that this model mainly reflects the systemic pathogenic potential of the isolate under experimental conditions and does not reproduce the local progression from otitis media to intracranial infection observed in the alpaca. Intraperitoneal inoculation bypasses the local barriers of the middle ear and adjacent tissues. Therefore, this model cannot be used to assess the neurovirulence or brain abscess-forming ability of CS234. Future studies could use local middle-ear or auricular inoculation models in rodents, combined with pathological and bacteriological examination of the ear, adjacent tissues, and intracranial tissues, to more directly evaluate local spread and brain abscess formation.

The antimicrobial resistance profile of CS234 was another important finding of this study. Disk diffusion was used for broad antimicrobial susceptibility screening, and the isolate showed resistance to several antimicrobial classes. However, minimum inhibitory concentration (MIC) testing was not performed, which limited quantitative assessment of the resistance level. For antimicrobial agents without applicable interpretive criteria for *M. sciuri*, inhibition-zone diameters could only be reported as phenotypic observations and could not be reliably classified as susceptible or resistant. Future testing of representative antimicrobial agents using standardized MIC methods would provide a more accurate description of the resistance phenotype and facilitate comparison between resistance genotypes and phenotypes.

The results of resistance gene detection were not fully consistent between PCR and whole-genome analysis. PCR screening produced amplicons of the expected sizes for *aacA-aphD*, *tetK*, and *mphC*, but the corresponding resistance determinants were not identified by CARD or ResFinder. Because PCR and database-based whole-genome analysis rely on different detection principles, the specific reason for this discrepancy remains unclear. In addition, minocycline showed an intermediate phenotype, but genomic analysis did not identify a known resistance determinant that could clearly explain this phenotype. The molecular basis of this intermediate phenotype therefore remains unclear.

In contrast, the genomic evidence for *mecA* was more robust. The complete *mecA* sequence was supported by both ResFinder and CARD and was located within a class A mec gene complex consisting of *mecA*, mecR1, and mecI. However, this region could not be assigned to a complete known SCCmec type. The presence of *mecA* provides a genetic basis for β-lactam resistance in CS234, but gene presence alone does not establish the corresponding resistance phenotype. In this study, the oxacillin inhibition-zone diameter was 17 mm. Because no species-specific disk diffusion breakpoint is available for *M. sciuri*, this result was not categorized as susceptible or resistant. Therefore, the current disk diffusion result is insufficient to determine the relationship between the *mecA* genotype and the oxacillin phenotype. Further MIC testing of β-lactam antimicrobial agents, together with assessment of *mecA* expression when necessary, would provide a more direct evaluation of the relationship between the *mecA* genotype and the resistance phenotype in this isolate.

Recent reports have also described human clinical infections associated with *M. sciuri*. A case of peritonitis caused by *M. sciuri* was previously reported in a patient undergoing peritoneal dialysis [38]. In addition, *M. sciuri* has been isolated from clinical samples from patients with surgical site infections and from the urine of a patient in China [39,40]. These reports indicate that *M. sciuri* can occur in human clinical settings. However, the present study included no human isolates and did not investigate epidemiological links between animals and humans. Therefore, zoonotic or cross-species transmission cannot be inferred from this case. Comparative genomic and epidemiological studies of isolates from different host sources are needed to further assess its potential for cross-host transmission.

The main limitations of this study relate to the available case material and the small sample size. Because well-preserved intracranial lesion tissues were unavailable, histopathological examination could not be performed, and the bacteria could not be directly localized within the intracranial lesions by immunohistochemistry or in situ hybridization. In addition, other potential bacterial and fungal pathogens were not systematically screened by molecular methods. Therefore, postmortem contamination or the involvement of other pathogens cannot be completely excluded. For this reason, the recovery of bacteria from multiple affected sites together with molecular identification was interpreted as supporting a strong etiological association rather than establishing a definitive causal relationship. Another limitation is that this study involved only one alpaca and one bacterial isolate. Therefore, the prevalence or infection frequency of *M. sciuri* in alpacas cannot be estimated from this case, and the characteristics of CS234 may not represent those of other alpaca-derived isolates. Future epidemiological studies should include more alpacas from different herds and geographic regions. Sampling should include both healthy animals and animals with otitis media or other suppurative infections. Antimicrobial susceptibility testing and whole-genome sequencing of multiple isolates would help evaluate the epidemiological characteristics and genetic diversity of *M. sciuri* in alpacas.

For clinical practice, this case suggests that zoos and alpaca farms should strengthen case documentation when persistent or recurrent otitis media, suppurative infections, or suspected multidrug-resistant bacterial infections occur. Bacterial culture and antimicrobial susceptibility testing should be performed whenever feasible to guide more targeted antimicrobial selection. Hand hygiene and appropriate cleaning and disinfection of shared equipment and the environment remain basic measures for reducing the spread of opportunistic pathogens. For multidrug-resistant isolates that recur, occur in clusters, or have clear epidemiological links, whole-genome sequencing may be used to compare genetic relatedness among isolates and to investigate resistance-associated mobile genetic elements. Integrating clinical case information, antimicrobial use, susceptibility results, and genomic data may provide a more reliable basis for antimicrobial resistance surveillance and infection control in alpaca farms and zoos.

Overall, this case supports a strong etiological association between *M. sciuri* and otitis media with intracranial suppurative lesions in this alpaca. It also expands current knowledge of the antimicrobial resistance and potential virulence-associated genetic features of alpaca-derived *M. sciuri*. Epidemiological, functional, and comparative genomic studies involving additional clinical cases and isolates will help further clarify the clinical significance of *M. sciuri* in alpacas and its epidemiological relevance across host species.

## 5. Conclusions

This study identified *Mammaliicoccus sciuri* CS234 in an alpaca with chronic otitis media and an intracranial abscess. The available bacteriological and molecular evidence supports a strong etiological association between CS234 and the suppurative disease in this case, although a definitive causal relationship cannot be established. CS234 showed resistance to multiple antimicrobial classes, and PCR screening detected several putative virulence-associated targets. Notably, the identity of *exhB* was confirmed by bidirectional Sanger sequencing and, to our knowledge, has not previously been reported in *M. sciuri*. The intraperitoneal mouse challenge supports the systemic pathogenic potential of this isolate; however, this model does not demonstrate neurovirulence or the ability to form brain abscesses. For persistent or recurrent otitis in alpacas, particularly in cases accompanied by neurological signs, bacterial culture and antimicrobial susceptibility testing should be performed before antimicrobial treatment whenever feasible. Treatment decisions should consider antimicrobial susceptibility results, tissue penetration, and animal safety. Zoos and alpaca farms should strengthen surveillance of recurrent otitis and multidrug-resistant infections, promote prudent antimicrobial use, and reinforce basic biosecurity measures, including hand hygiene and appropriate cleaning and disinfection of shared equipment and the environment. Larger clinical and epidemiological studies, comparative genomic analyses, and functional studies of *exhB* are needed to further clarify the clinical significance of *M. sciuri* in alpacas.

## Figures and Tables

**Table 1:** Primers for PCR amplification of antimicrobial resistance genes.

Primer Name	Primer Sequence (5′-3′)	Annealing Temperature (°C)	PCR Product (bp)
mecA1-F	TTGAAGAAGCAACAACGCAC	53	298
mecA1-R	GAACCGTAGTCATCTTTCATGTTG		
mecC-F	GGATCTGGTACAGCATTACAACC	53	598
mecC-R	TGCTTTAAATCRATMTTGCCG		
aacA-aphD-F	TAATCCAAGAGCAATAAGGGC	51	227
aacA-aphD-R	GCCACACTATCATAACCACTA		
ermA-F	AAGCGGTAAACCCCTCTGA	54	190
ermA-R	TTCGCAAATCCCTTCTCAAC		
ermC-F	AATCGTCAATTCCTGCATGT	51	840
ermC-R	TAATCGTGGAATACGGGTTTG		
tetK-F	GTAGCGACAATAGGTAATAGT	55	360
tetK-R	GTAGTGACAATAAACCTCCTA		
mecA-F	GTAGAAATGACTGAACGTCCGATAA	53	310
mecA-R	CCAATTCCACATTGTTTCGGTCTAA		
aadD-F	CAAACTGCTAAATCGGTAGAAGCC	56	296
aadD-R	GGAAAGTTGACCAGACATTACGAACT		
aphA3-F	GGCTAAAATGAGAATATCACCGG	52	523
aphA3-R	CTTTAAAAAATCATACAGCTCGCG		
blaZ-F	ACTTCAACACCTGCTGCTTTC	53	240
blaZ-R	TGACCACTTTTATCAGCAACC		
msr-F	GGCACAATAAGAGTGTTTAAAGG	51	940
msr-R	AAGTTATATCATGAATAGATTGTCCTGTT		
mphC-F	GAGACTACCAAGAAGACCTGACG	55	722
mphC-R	CATACGCCGATTCTCCTGAT		

**Table 2:** Primers used for PCR amplification of virulence-associated genes.

Primer Name	Primer Sequence (5′-3′)	Annealing Temperature (°C)	PCR Product (bp)
HLD-F	AAGAATTTTTATCTTAATTAAGGAAGGAGTG	56	111
HLD-R	TTAGTGAATTTGTTCACTGTGTCGA		
PVL-F	TATTGGTGATGGCGCTGAGG	59	598
PVL-R	ACCACTGTGTACTAATGGGGG		
HLA-F	AGCGTTAATGCTGCACCTAA	52	608
HLA-R	GCGTTACTATCCGTATTTGGTT		
clfA-F	GCGTTACTATCCGTATTTGGTT	53	272
clfA-R	TTGTACTACCTATGCCAGTGTC		
HLB-F	GGTACTTGGCCGTTCCACTT	57	840
HLB-R	TTGACCTACTGAGCAGCGTG		
coa-F	ATAGAGATGCTGGTACAGG	52	1586
coa-R	GCTTCCGATTGTTCGATGC		
HLG-F	GTCAYAGAGTCCATAATGCATTTAA	53	535
HLG-R	CACCAAATGTATAGCCTAAAGTG		
fib-F	CTACAACTACAATTGCCGTCAACAG	52	404
fib-R	GCTCTTGTAAGACCATTTTCTTCAC		
fnbA-F	GTGAAGTTTTAGAAGGTGGAAAGATTAG	52	543
fnbA-R	GCTCTTGTAAGACCATTTTTCTTCAC		
fnbB-F	GTAACAGCTAATGGTCGAATTGATACT	52	524
fnbB-R	CAAGTTCGATAGGAGTACTATGTTC		
agr-F	TAACATTTAATAAGAATCAA	45	292
agr-R	GTCACAAGTACTATAAGCTGCGAT		
ExhA-F	ATAGAGGAGAAATCAACATG	44	316
ExhA-R	CTATAGTTACTTGACCTCTA		
ExhB-F	GACCATGACTATCACTCTAT	46	464
ExhB-R	GAGAACGATTCTCGTAAAAT		
ExhC-F	GAATAAATATTATGGAGTCTCTCCTGATC	50	525
ExhC-R	CCATAGTATTTCAATCCAAAATCAGTAC		
tsst-1-F	GCTTGCGACAACTGCTACAG	54	559
tsst-1-R	TGGATCCGTCATTCATTGTTAT		
cap5-F	ATGACGATGAGGATAGCG	52	306
cap5-R	CTCGGATAACACCTGTTGC		

**Table 3:** Whole-genome characteristics of *M. sciuri* CS234.

Characteristic	Value
Genome size	2,870,194 bp
GC content	32.82%
Number of contigs	2
Contig N50	2,842,952 bp
Assembly gaps	0
Genome coverage	100%
Mean sequencing depth	329.42×
CheckM completeness	99.45%
CheckM contamination	2.39%
BUSCO completeness	100%
Protein-coding genes	2764
rRNA genes	19
tRNA genes	57
ANI with *M. sciuri* NCTC12103	95.58%
GenBank accession	CM152820.1

**Table 4:** Biochemical characteristics of CS234 compared with the typical profile of *M. sciuri*.

Item	CS234	Typical *M. sciuri* Profile
Glucose	+	+
Maltose	+	Variable
Mannitol	+	+
Sucrose	+	+
Lactose	−	Variable
Hydrogen sulfide production test	−	NR
Citrate test	+	NR
Indole test	−	NR
V-P test	−	−
MR test	−	NR

Note: +, positive; −, negative; Variable, variable reaction among strains; NR, not reported in the cited reference [28].

**Table 5:** Antimicrobial susceptibility profile of *M. sciuri* CS234.

Abbreviation	Drug Name	Content/Tablet	Inhibition Zone (mm)	R/I/S/NI
P	Penicillin G	10 units	28	R
OX	Oxacillin	10 μg	17	NI
AMP	Ampicillin	10 μg	27	NI
CEX	Cephalexin	30 μg	25	NI
RAD	Cefradine	30 μg	19	NI
CXM	Cefuroxime	30 μg	20	NI
CAZ	Ceftazidime	30 μg	6	NI
CRO	Ceftriaxone	30 μg	16	NI
AK	Amikacin	30 μg	19	NI
GM	Gentamicin	10 μg	10	R
K	Kanamycin	30 μg	9	NI
N	Neomycin	30 μg	19	NI
TE	Tetracycline	30 μg	6	R
DX	Doxycycline	30 μg	9	R
MI	Minocycline	30 μg	16	I
E	Erythromycin	15 μg	0	R
MID	Midecamycin	30 μg	22	NI
VA	Vancomycin	30 μg	26	NI
NOR	Norfloxacin	10 μg	0	NI
CPFX	Ciprofloxacin	5 μg	34	S
PB	Polymyxin B	300 IU	18	NI
C	Chloramphenicol	30 μg	26	S
CC	Clindamycin	2 μg	33	NI

Note: S, susceptible; I, intermediate; R, resistant; NI, not interpreted. Categorical interpretations were based on CLSI M100, 33rd edition (2023), using *Staphylococcus* spp. criteria as surrogate criteria where applicable. For antimicrobial agents without applicable disk diffusion criteria, only inhibition zone diameters are reported.

**Table 6:** Mortality of ICR mice after intraperitoneal challenge with CS234.

Group	Dose (CFU/Mouse)	Equivalent Dose (CFU/g Body Weight)	Total (*n*)	Deaths (*n*)
D1	2.00 × 10^8^	1.00 × 10^7^	10	0
D2	3.18 × 10^8^	1.59 × 10^7^	10	1
D3	5.02 × 10^8^	2.51 × 10^7^	10	2
D4	7.96 × 10^8^	3.98 × 10^7^	10	4
D5	1.26 × 10^9^	6.31 × 10^7^	10	7
D6	2.00 × 10^9^	1.00 × 10^8^	10	9

Note: Equivalent CFU/g body weight values were calculated based on a standard body weight of 20 g per mouse.

## Data Availability

The original contributions presented in this study are included in the article. Further inquiries can be directed to the corresponding authors.
